# Race Analysis in Competitive Swimming: A Narrative Review

**DOI:** 10.3390/ijerph18010069

**Published:** 2020-12-24

**Authors:** Tomohiro Gonjo, Bjørn Harald Olstad

**Affiliations:** Department of Physical Performance, Norwegian School of Sport Sciences, 0863 Oslo, Norway; b.h.olstad@nih.no

**Keywords:** performance analysis, competition analysis, race segments, start, clean swimming, turn, finish

## Abstract

Researchers have quantified swimming races for several decades to provide objective information on race strategy and characteristics. The purpose of the present review was to summarize knowledge established in the literature and current issues in swimming race analysis. A systematic search of the literature for the current narrative review was conducted in September 2020 using Web of Science, SPORTDiscus (via EBSCO), and PubMed. After examining 321 studies, 22 articles were included in the current review. Most studies divided the race into the start, clean swimming, turn, and/or finish segments; however, the definition of each segment varied, especially for the turn. Ideal definitions for the start and turn-out seemed to differ depending on the stroke styles and swimmers’ level. Many studies have focused on either 100 m or 200 m events with the four strokes (butterfly, backstroke, breaststroke, and freestyle). Contrastingly, there were few or no studies for 50 m, long-distance, individual medley, and relay events. The number of studies examining races for short course, junior and Paralympic swimmers were also very limited. Future studies should focus on those with limited evidence as well as race analysis outside competitions in which detailed kinematic and physiological analyses are possible.

## 1. Introduction

In competitive sports, it is important for athletes and coaches to understand the behavior of the athletes and its relation to the competition results to improve their future outcomes. For this reason, performance analysis provides an essential role to support the development of athletes from a scientific perspective. Performance analysis is defined as objective analysis of actual sports performance without involving self-reports or a laboratory setting [1]. In swimming, performance analysis involves a greater challenge compared with on-land sports as any physical equipment, such as a set of small reflective markers, increases hydrodynamic drag [2], and thereby makes the investigation far from ‘actual’ swimming performance analysis. 

One of the few performance analysis methods in swimming is the race analysis, which is the investigation of races in competitions [3] or simulated races following the official race regulations [4]. Race analyses in swimming are generally conducted using official race results, including the reaction time, lap time, and finishing time, often combined with video footage. Official race results are useful when pacing strategy of swimmers are assessed [5], whereas video footage is essential when detailed race information (such as the duration of start, clean swimming, turn, and finish during the race) is required. 

The official race rules regulated by Fédération Internationale de Natation (FINA) have been constantly updated. Some regulation changes in the last three decades were relevant to multiple swimming strokes, such as the restriction of over 15 m of underwater swimming after the start and each turn. On the other hand, other regulation updates were specifically applied to a single stroke, e.g., the permission of exerting one dolphin kick during the breaststroke underwater segment. Such changes directly affect required skills and race strategy of swimmers, and therefore it is of great importance to constantly investigate races to update the understanding of relative importance of different skills; for instance, start, clean swimming, turn, and finish [3,6,7]. This also means that coaches and athletes should be careful when applying scientific race analysis knowledge into practice as information obtained under old competition rules might not be applicable to the current competitive swimming races. Summarizing race analysis studies that have been conducted to date would provide insights into what is important in current swimming race analysis and which race skills should be further investigated. 

It should also be noted that available race analysis studies are not consistent in their analytical methods. For example, some studies have defined the turn segment as from the 5 m before the wall until the 15 m after the wall push-off [3,6], while other studies have considered the same segment as 7.5 m before and after the wall [7,8]. This means that readers should always be aware of the methodological background and limitation of each research and its difference from other studies. Similarly, research outcomes should also depend on the analyzed group(s) of swimmers (i.e., international or regional level, male or female, junior or senior swimmers, Olympic or Paralympic athletes), which should also be considered when interpreting race analysis results. 

Due to the inconsistency in the methods as well as a wide range of analyzed groups in the literature and frequent rule changes, it is likely that results reported in swimming race analysis studies vary. Summarizing the extant literature on this topic and identifying the similarities and differences among studies would be useful to understand how the race analysis at/with different time/methods would affect the outcomes, which would consequently contribute to a better understanding of how the race analysis knowledge should be applied to practice. Therefore, the purpose of the current paper was to provide a review on swimming race analysis topic, with a specific focus on biomechanical (spatiotemporal) assessment, to highlight the knowledge established in the literature and current issues in the field.

## 2. Materials and Methods 

A systematic search of online databases for the current narrative review was conducted in September 2020 to identify relevant research publications using Web of Science, SPORTDiscus (via EBSCO) and PubMed. The search terms used were ‘swimming’ and ‘race’ with an additional keyword of ‘race analysis’, ‘competition analysis’, ‘Olympics’, or ‘World Championships’ with only English written articles published in peer-reviewed journals being included in the initial records. As there is currently no literature review available on spatiotemporal swimming race analysis topic, no limit was set for the publication year during the search process. From identified publications, studies that met one or more of the following criteria were excluded; (i) studies that focused on sports other than competitive swimming or analyzed Masters swimming races; (ii) papers that did not use races in actual competition or a simulated race; (iii) studies published in academic journals corresponding to conference proceedings (e.g., journal supplements); (iv) articles that focused on ‘pacing’, as this topic has recently been reviewed elsewhere [5,9]; (v) studies in which only the ‘block time’ or ‘reaction time’ was considered due to its small contribution on the total race time; (vi) research that only analyzed stroke cycle skills, such as stroke frequency and length, rather than swimmers’ behavior throughout a race segment(s). As only searching electric databases likely miss some relevant studies, reference lists of the remaining articles were carefully checked, and English-written journal articles that did not meet any of the exclusion criteria were also included [10]. 

## 3. Results and Discussion

### 3.1. Overall Information on the Reviewed Literature

**Figure 1:**
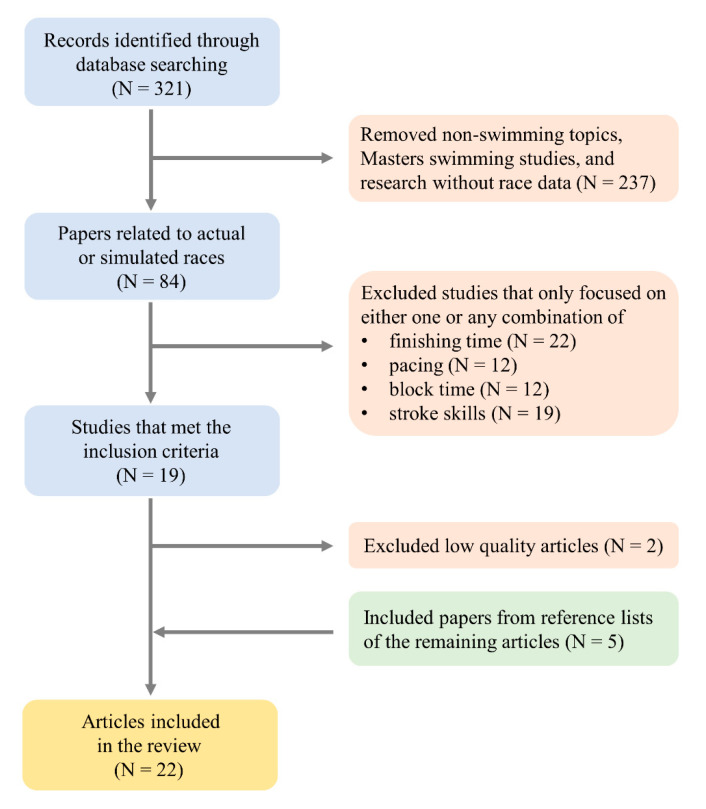
Flow chart summary of the study selection process.

The initial database search identified 321 records that were relevant to the search keywords. The record screening based on the exclusion criteria resulted in a total of 19 journal articles remaining at the end of the process. Among the 19 articles, two articles were excluded due to insufficient quality of data or explanation making the interpretation of the results impossible. From the reference lists of the 17 articles, five articles were added to the selected items that resulted in a total of 22 articles included in the present review (Figure 1).

**Figure 2:**
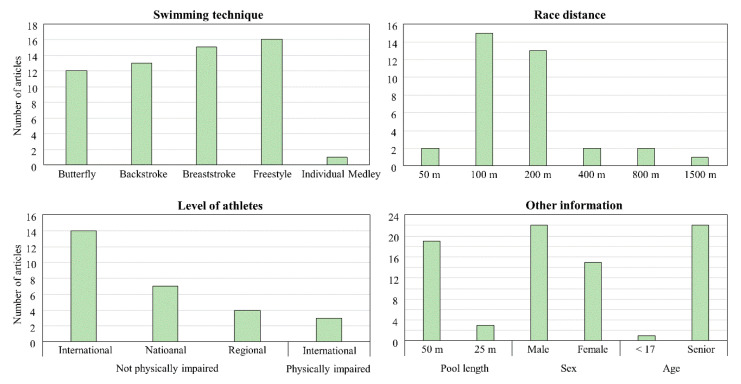
Summary of the key information extracted from the reviewed articles.

All reviewed studies analyzed a race as a sequence of all or either of the following events; the beginning of the race, the segment where swimmers only perform whole-body swimming, a phase around the turn, and the end of the race. Even though researchers have used different names for those segments, those segments are defined as the start, clean swimming, turn, and finish in the current review for consistency. Summary of the reviewed articles is given in Figure 2. The most investigated stroke, distance, and swimmers’ level were freestyle, 100 m, and international physically non-impaired swimmers, respectively. Most of the studies focused on long course races with senior groups, and the number of papers that assessed male swimmers was larger than those investigated female swimmers. 

The majority of articles reviewed in the current study focused on multiple events, with six exceptions [4,8,11,12,13,14] in which only a single event was investigated. Among the studies that investigated several events, two studies analyzed different distances in a single stroke [15,16], one study investigated a single stroke with a single distance but with different levels of visual impairment [17], and the others assessed all four strokes [3,6,7,18,19,20,21,22,23,24,25,26]. There was only one study that investigated individual medley events [25], and medley events have not been analyzed for more than 30 years after that study. Only one study focused on a short course competition (which only explored one event—200 m backstroke [8]). Two studies [4,11] examined short course races, but not during real competitions. No study evaluated young swimmers’ races during competitions; however, one study [11] investigated simulated 50 m freestyle races for a wide range of ages (9–22 years old). Among physically not impaired athletes, international swimmers [3,6,12,13,14,15,16,17,19,21,24,25,26,27] were the most investigated group, followed by national [4,7,8,13,14,16,22] and regional [8,11,18,22]. Three studies [17,20,23] analyzed races for Paralympic athletes. No race analysis studies were identified for any relay events. 

Despite the common practice of dividing a race into the start, clean swimming, turn, and finish segment, the definition of these segments varied among studies (Table 1). This is particularly noticeable in the turn segment, in which seven different definitions were identified among the literature. The most consistent race segment was the clean swimming segment, which is almost always deined as the part of the race that is not included in other segments (such as the start, turn, and finish), except one research [12]. The finish was the least investigated segment.

### 3.2. Variation in Race Segment Definitions

Even though a race has been divided into three to four segments, we identified varying definitions for each segment. The wide range of variability suggests that coaches and researchers should be careful when interpreting the results and compare them with those of their own swimmers or other studies.

#### 3.2.1. Start Segment

Most of the reviewed studies defined the start segment as from the start signal until the swimmer’s head reached the first 15 m point. This is reasonable since the FINA regulations restrict the underwater locomotion to 15 m from the wall in most strokes (except breaststroke), meaning that swimmers should start the clean swimming motion at or before this point. There were two studies that defined the start segment as the first 10 m instead of 15 m [15,23], both of which had been conducted before the 15 m definition became common for the start segment.

A possible explanation for the switch of the definition from 10 m to 15 m might be related to a change in the race strategy. Approximate differences in the breakout distance between 1984 and recent years are shown in Table 2 and Table 3. In 1984, swimmers performed their breakout in the start segment before they reached 10 m in freestyle, backstroke, and butterfly races [25]. The authors of the 1984 study presented hand displacement when swimmers completed the first stroke cycle, rather than the head displacement, as the breakout distance. Therefore, the exact location of the head breakout was unknown. However, it was likely that the breakout distance in breaststroke was also before or around 10 m given that the completion of the first stroke was around 10–12 m and the stroke length of those swimmers was around 1.4–1.75 m/cycle [28]. Thus, 10 m start segment definition was reasonable in the early days.

At present, however, swimmers perform their breakout much later. Table 2 and Table 3, which are based on five studies among the reviewed literature [3,6,18,19,25], exhibit a great improvement in the breakout distance in all events. This is especially the case in backstroke events where the distance is now 49–66% longer compared with races in 1984. As described above, the breakout distance in 1984 was likely much shorter than the exhibited distance, meaning that the increase in the breakout distance from the past to the present is even greater than the estimated improvement. In many events, the breakout distance is between 10 and 15 m that justifies the use of 15 m distance as a criterion of the start segment.

However, depending on swimmers’ level, 10 m might still be informative as the start segment definition in some races. Three studies show that international level swimmers breakout from the water after 10 m regardless of the gender and stroke [3,6,19]. Therefore, it is likely that the 15 m definition is preferable over the one with 10 m when investigating international level swimmers (at least in 100 and 200 m events). However, in a study conducted in 2014 [18], regional and national level swimmers surfaced from the water before 10 m in both male and female freestyle events, and this was also the case in female regional level butterfly swimmers [18]. These results imply that researchers and coaches might have to define the start segment carefully depending on the level of investigated swimmers when they employ a fixed-distance method.

In contrast to the studies utilizing fixed-distance definitions, others defined the start segment based on the individual breakout point of the athletes. The first study that used the individualized definition among the reviewed articles was published in 1984 [25]. In that study, researchers defined the start segment as from the starting signal to the completion of the first arm stroke after the breakout. Interestingly, it was almost 30 years after the first study when another study [7] applied a start segment definition based on the individual breakout point. The reason for the unpopularity of the individualized race analysis for a long period of time is unclear, but there are some potential explanations.

The first potential reason is a standardization of the comparison. Since swimming is a time-trial sport that requires athletes to complete a given distance in the least time possible, it is straightforward to define each race segment using the same distance. This process makes a comparison between different swimmers and events easier than individually different segment distance in which researchers should consider not only a difference in time but also in the distance and mean velocity.

The second possibility is the equipment used for data collection. In the study from 1984 [25], a cine camera was used to quantify segmental outcomes, while in later studies, video cameras have been commonly used. Despite the portability and low cost, video cameras in the early days were inferior to cine cameras in many features such as the image resolution and a range of shutter speed [29]. Perhaps investigating the start segment using the fixed-distance method was more suitable than focusing on the individual strategy due to the limited quality of the video images. This could explain why the individual-based race analysis has gained its popularity in the last several years, as the recent video cameras are capable of recording images with much higher quality compared with those in the early days.

#### 3.2.2. Turn (Turn-Out) Segment

Similar to the start segment, one big difference between turn segment definitions was whether the definition was based on fixed-distance or the individual breakout point. However, a notable difference from the start segment was a large variety in each group of definitions (fixed- or individual-distance). Among the fixed-distance methods, three different definitions were identified. A point in common between the three fixed-distance definitions is that all three methods consider the segment consisting of turn-in and turn-out.

A widely used and the oldest fixed-distance definition among the reviewed studies is 7.5 m turn-in and -out. The 7.5 m turn-out definition seemed reasonable at least in the early days. In 1984, it was reported that swimmers showed the breakout before 7 m in most of the male and female events, except for breaststroke in which the breakout distance was between 7 and 8.5 m depending on the sex and distance of the race (Table 4 and Table 5) [26]. As discussed in the start segment section above, it should be noted that the researchers in 1984 also defined the turn-out distance at the instant of the first arm cycle completion, meaning that the breakout distance should have been slightly shorter than the reported distance. Therefore, it was likely that the breakout distance in breaststroke was also before, or at least close to 7.5 m.

However, 7.5 m definition is not likely adequate at present as the breakout distance is considerably longer in all swimming strokes, as is the case for the start segment. The breakout distance after the turn between 1984–2020 is shown in Table 4 and Table 5 [3,6,18,19,26]. The summary of those studies clearly exhibits that the breakout distance has increased in all 100 and 200 m events. A 7.5 m turn-out definition might be still applicable at present in freestyle events in which many swimmers break the water surface before they reach 8 m from the wall. However, researchers and performance analysis should be aware that 7.5 m distance cannot define the turn-out in many events.

In breaststroke events, 10 m after the wall push-off seems to represent the turn-out segment well, as swimmers break out from the water at almost exactly 10 m in male events and between 7.5 and 10 m in female events in the four recent studies. Furthermore, Olstad et al. [4] conducted a detailed race analysis in breaststroke and also reported that elite breaststroke swimmers showed breakout around 8.9–9.4 m after the push-off in a 100 m short course race.

In butterfly and backstroke, the breakout distance after the turn varies more compared with the other two strokes, especially among different levels of swimmers. Table 4 and Table 5 show that international level swimmers generally travel a longer underwater distance than regional and national level swimmers. The difference between international and regional or national level swimmers have not been established; however, Veiga et al. [18] compared regional and national level swimmers and reported that national level swimmers travelled longer before the breakout compared with regional swimmers in butterfly and backstroke events. This evidence supports the effect of the competitive level on the breakout distance in the two strokes, and this means that different criteria might be necessary depending on the level in butterfly and backstroke when using a fixed-distance method. For example, in male 100 m butterfly and backstroke events, 10 m turn-out definition might be better than 15 m when investigating regional swimmers, but the opposite is likely the case when the research interest is with international swimmers.

#### 3.2.3. Turn (Turn-In) and Finish Segments

In both fixed-distance and individual-based segment methods, there seems to be no concrete agreement in the method of defining the beginning of the turn segment. Studies that employed the fixed-distance method defined the beginning of the turn-in as either 7.5 m [7,13,14,15,16,17,23] or 5 m [3,4,6,12,27] before the wall. However, no study has presented a rationale for this decision. This is probably due to the lack of knowledge in motor control aspects of swimming turns. Swimming turns have been analyzed descriptively (such as studies reviewed in the present study) or in details with biomechanical equipment (e.g., force platform and underwater cameras [30]). However, it is currently unknown how swimmers adjust their swimming motion toward the end of each lap to prepare for the following turning motion.

The lack of knowledge is also a likely reason for the inconsistency in the individual-based turn definition. Some studies [7,8,18,20,22] considered a short period of motion (e.g., from the last hand entry to the wall contact) as a part of the turn segment, while other studies only investigated the phase from the wall contact to the head emersion [19,21,24] or mixed these two strategies depending on the stroke [26]. Nevertheless, in both cases, it seems that the effect of swimming motion adjustment for the turn was assumed to be almost negligible.

This is also the case for the finish segment—there is a lack of knowledge in how swimmers control their motion to perform the finishing touch in the manner that minimizes the time required for the last part of the race. There were eight studies that investigated the finish segment among the reviewed literature; five defined the segment as the last 5 m [4,6,13,16,17], two studies assumed it as the last 7.5 or 10 m [15,23], and one did not specify the definition [11]. Even though the majority of them seem to agree on defining the finish segment as the last 5 m of the race, it does not necessarily mean it is a reasonable choice, as this is rather a custom than an evidence-based method.

In future studies, it would be beneficial to identify how many arm stroke cycles swimmers generally use to adjust the distance between the body and the wall to perform a turn or the finish. As an example, analyzing the stroke length and frequency for every stroke cycle in each lap and assessing their change when approaching the wall would be useful as the first step to answer this question.

#### 3.2.4. Clean Swimming Segment

Almost all reviewed studies defined the clean swimming segment as parts of the race that did not belong to the start, turn, and finish segments. As discussed in the previous sections, there has been a wide range of variability in the start, turn, and finish segments, which consequently means there has also been large variability in the clean swimming distance. Veiga et al. [7] compared the fixed-distance and individual-based method and reported that the fixed-distance method underestimated both the distance travelled and the mean velocity during the clean-swimming segment, showing the impact of the segment definition on the outcomes.

A unique clean-swimming segment definition was used by Morais et al. [12] in which the researchers assessed 15–35 m of each lap as the clean swimming to minimize the effect of the wall push-off and the approach to the wall in long-course 800 m freestyle races. Considering that the velocity, stroke frequency and length derived from 25–35 m are not different from those obtained from a distance between breakout and 15 m in the second lap in 100 freestyle races [21], it is probably reasonable to assume that the effect of the turn is negligible after 15 m, at least in freestyle events. Even though the effect of turning on the subsequent swimming segment in long-distance events has not been investigated, it is unlikely that one would observe a stronger effect in long-distance events than 100 m races, since swimmers break out from the water around 5 m after turns [12] that is much shorter than 100 m events (Table 4 and Table 5). As discussed above (Section 3.2.3), it is currently unknown how many meters or numbers of stroke cycles swimmers need to adjust their swimming motion to prepare for turns. However, Veiga et al. [21] suggest that swimmers maintain a stable velocity, stroke frequency and length at least until 35 m after the turn (in 100 m races); therefore, 15–35 m definition seems reasonable.

Regardless of the definitions used, knowledge obtained for the clean-swimming segments is relatively limited compared with the start and turn segments. Many studies have reported the average velocity, stroke frequency and length in the clean swimming segment, which provide information on the stroke cycle skills of investigated swimmers. However, it is currently unclear how kinematics varies stroke-to-stroke, except for front crawl stroke [31,32]. Inter-stroke velocity fluctuation during a race would especially be an interesting topic to explore, as it is widely accepted that a stable velocity would require low energy expenditure [33].

### 3.3. Differences between Swimming Events

Among the reviewed literature, no study analyzed 50 m butterfly, backstroke, and breaststroke, which was probably due to those three sprint events not being included in the Olympic games. Instead, many studies have analyzed the four swimming strokes in 100 and 200 m. The focus of the different studies varies, but one common analysis is to investigate differences between events (between strokes or distances).

Differences and similarities between the four strokes were highlighted by Morais et al. [3], in which the researchers compared a number of start and turn variables between the four strokes in both males and females. Similarities found between the strokes in both male and female swimmers included; 15 m time between freestyle and butterfly, total turn time (5 m turn-in and 15 m turn-out) between butterfly and backstroke, 5 m turn-in time between backstroke and breaststroke and between freestyle and butterfly, breakout time after the turn among all strokes apart from freestyle, the % contribution of 15 m time to the finishing time between backstroke and freestyle and between breaststroke and butterfly, as well as the % contribution of breakout time after the turn among butterfly, backstroke, and breaststroke.

However, compared with the study by Morais et al. [3], a study by Veiga et al. [22] presented a smaller number of similarities among the strokes. In the latter study, the researchers showed similarities only in the underwater distance between butterfly and backstroke and the velocity of an approach phase (the period from the last stroke to the wall contact) between backstroke and freestyle among the four strokes in 200 m race turns. The contrast in the results presented in the two studies might suggest that the differences between the strokes in the turn segment are more evident in 200 m than in 100 m events. Nevertheless, from those two studies and another research that investigated the underwater and surface strategies in 200 m races [24], it is evident that swimmers have the shortest breakout distance after the turn in freestyle among the four strokes and the total turn time is the fastest in freestyle than the other three strokes [3,22]. Detailed statistical results in 200 m start segments have not been reported in the reviewed studies, and the evidence underpinning differences in the start segment between the strokes is limited to 100 m results reported by Morais et al. [3]. However, judging from data presented in the reviewed studies (Table 1 and Table 2), it seems that the breakout distance in 200 m freestyle is also shorter than the other three strokes.

Some researchers have also investigated the effect of the race distance on the segmental parameters. Arellano et al. [15] investigated 50, 100, and 200 m freestyle races and showed that finishing time in all distances exhibited consistent positive relationships with the start, turn, and finish segment times as well as negative relationships with clean swimming velocity and stroke length. A similar study was conducted later but in 100 and 200 m breaststroke swimming [16], in which similar trends (such as positive relationships between the finishing time and segment times) and faster time in all segments in 100 than 200 m race were observed. However, a recent study has observed a different result. Marinho et al. [6] compared 100 and 200 m races in the four swimming strokes and found that male breaststrokers spent similar 15 m time in both 100 and 200 m races. Given that female swimmers showed faster 15 m time in 100 m than in 200 m breaststroke and many variables (underwater distance and speed as well as breakout distance and time) exhibited differences between 100 and 200 m breaststroke races in the same study, the similar 15 m result between the distance might be due to the type II error, but it is also possible that the difference reflects changes in the start technique in the last 20 years.

The results in the other three strokes reported in the study by Marinho et al. [6] were in line with other studies [15,16]. Here swimmers showed a faster time in both the start (from the start signal to 15 m) and turn (5 m turn-in and 15 m turn-out) segments in 100 m than 200 m races apart from female backstroke start time (yet, the mean value was slightly larger in 200 m). However, underwater distance and time were similar between the distances in many cases except for the underwater time in male butterfly turns. These outcomes indirectly suggest that swimmers might put a similar amount of effort into the underwater locomotion in 100 and 200 m races, at least in freestyle and backstroke.

The results in the finish segment reported by Marinho et al. [6] also had a different trend from the early studies [15,16]. Both early studies reported that the finish segment time increased as the race distance extended. However, in the latest study by Marinho et al. [6], this trend was only observed in butterfly (both in males and females) and breaststroke (only in females). As introduced in an earlier section, the finish segment is not generally considered in recent race analyses employing the individual-based method. However, assuming that the clean swimming velocity is different between event distances, the results presented by Marinho et al. [6] might raise a question on the neglect of the finish segment.

Knowledge in the individual medley race characteristics and their differences from those in other strokes is very limited. Among the reviewed literature, one study [25] analyzed 200 and 400 m individual medley races, but the analysis in that study was limited to only the start segment. In that study, the researchers showed identical start segment time and distance (defined as the end of the first arm cycle after the breakout) between 200 m individual medley (3.75 ± 0.44 s [male time], 3.63 ± 0.31 s [female time], 9.87 ± 0.74 m [male distance], and 8.79 ± 0.66 m [female distance]) and 200 m butterfly (3.71 ± 0.30 s [male time], 3.71 ± 0.44 s [female time], 9.93 ± 0.57 m [male distance], and 8.79 ± 0.80 m [female distance]), which implied comparable start skills between butterfly and individual medley swimmers. However, the study is from 1984, and swimming races in the present time are likely very different due to changes in rules and swimmers’ strategies, as discussed earlier. Therefore, race characteristics in current individual medley swimmers should be analyzed in future studies.

### 3.4. The Effect of Level and Sex on Swimming Races

Investigating the effect of swimmers’ level on each segment performance has also been a popular topic in race analysis. It has been reported that national level swimmers travel a longer underwater distance compared with regional level swimmers after the start and turns in butterfly, backstroke, and breaststroke (in 100 or 200 m events), but this is not the case in freestyle [18]. Given the shorter breakout distance in freestyle events than the other strokes discussed earlier in the present review, it is reasonable that the underwater distance does not have much effect on the finishing time in freestyle events. However, the results presented in this study [18] should be interpreted with caution, as the level of regional freestyle swimmers was considerably higher than those in the other three strokes, meaning that it was likely that some swimmers categorized as the regional group in freestyle events had a similar level as the national swimmers.

A similar study was conducted with international level swimmers [19], in which the relationships between the finishing time and the breakout distance as well as the mean underwater velocity were assessed in 100 and 200 m races. In 100 m events, the researchers found that breakout distance after the start and turn did not seem to have a large impact on the finishing time in most of the events (apart from the breakout distance after the butterfly start). It was suggested that maximizing the underwater velocity was more important than traveling a long distance underwater, at least in freestyle and backstroke. The researchers also highlighted different results in 200 m races. In all strokes, the breakout distance after the start and turn showed larger associations with finishing time compared with the underwater velocity in most cases. It should be noted that the results should not be generalized because the levels of analyzed athletes were very close to each other (finalists and semi-finalists in World Championships). Therefore, further studies are necessary to conclude the impact of the breakout distance and the underwater velocity on the finishing time. Nevertheless, despite the limitation, the sample quality of the study was valuable, and studies with a similar design should also be further conducted to obtain knowledge on world-class swimmers.

Two studies [8,14] compared the turn segment between different levels of swimmers in 200 m backstroke (short course) and butterfly (long course), respectively. Both studies analyzed the 7.5 m turn-in and -out and exhibited that high-level swimmers (such as international swimmers or finalists in a national competition) completed the turn segment in a shorter time compared with lower-level swimmers. A unique point in the 200 m butterfly study [14] was that not only the turn segment time during the race was investigated, but also kinematic variables that are difficult to obtain in competition race analysis (hand contact time and foot contact time) were analyzed using a separate protocol between preliminary heats and the final of the competition. They reported that the best swimmer had shorter foot and hand contact times compared with slower swimmers. The other study analyzed short course 200 m backstroke races [8] and displayed an interesting contrast with another study that investigated 200 m races in a long course competition [18]. The short course study [8] reported that the mean breakout distance after the turn of male swimmers with average FINA points of 718 ± 47.2 was 6.51 m, while the long course study [18] reported the mean breakout distance of 8.97 and 8.43 m for athletes with 753.5 ± 42.2 and 635.6 ± 39.5 FINA points, respectively. These two 200 m backstroke studies suggest that race strategies in short course and long course races are likely different. However, there have been few studies that analyzed short course races in real competitions, and it is required for researchers to investigate short course races in a wide range of events in future studies.

Differences between male and female swimmers have been discussed from the early days. Miller et al. [25] and Chow et al. [26] respectively compared the start and turn segment between the sexes. The former researchers reported longer underwater distance after the start in male than in female swimmers, despite the similar time spent. The latter study also showed the same results in the turn segment except for freestyle in which male swimmers travelled a longer underwater distance in a shorter duration than female. The distance and time results from these two studies also implied a faster underwater velocity in male than in female swimmers. A longer underwater distance and larger underwater velocity were also observed in males compared to females in a recent study [19], in which 100 and 200 m events for the four swimming strokes were analyzed. Another recent study [21] has reported that male swimmers show faster mean velocity in the ‘emersion’ phase (from the breakout to 15 m) compared with the ‘free-swimming’ phase (between 25 and 35 m) in both the first and second lap in 100 m races, but female swimmers exhibited the same tendency only in the first lap. However, this sex difference might be related to the short breakout distance from the wall in female swimmers, as the effect of the underwater locomotion on the emersion phase should have been stronger in male swimmers whose breakout distance was closer to 15 m than that in female swimmers.

Arellano et al. [15] compared male and female swimmers in three freestyle events and concluded that male swimmers swam faster in all segments (start, clean swimming, turn and finish) with a longer stroke length, but the stroke frequency was similar between males and females. Another finding by these authors included stronger relationships between the height and analyzed variables in male compared with female swimmers; however, this was not observed in breaststroke swimming [16]. In the last decade, individual strategies have gained attention. Veiga et al. [21], who investigated male and female 100 m races in World Championships, only observed significant relationships between finishing time and stroke frequency or length in female freestyle and backstroke, and male butterfly. In other words, in most of the events, swimming performance cannot be explained by a single factor, but elite swimmers optimize their stroke kinematics to maximize swimming velocity. A similar result was also found in a short course 100 m breaststroke study [4]. These studies suggest that the importance of individual strategies is particularly evident in the last decade.

To investigate individual strategies and obtain further insights into characteristics of different groups of swimmers, it is essential to assess race kinematics in detail. However, a limitation of current race analysis methods in competitions is that the camera setting is limited to only above-water view, meaning that detailed underwater kinematic information cannot be assessed in real competitions. Therefore, to better understand individual strategies during races, race analyses should be conducted not only during but also outside competitions, as has been done by some researchers [4,11,27].

### 3.5. Race Analysis in Paralympic Swimming

Some researchers have focused on Paralympic swimming races, albeit the number of studies is limited. The first detailed Paralympic swimming race analysis was conducted by Daly et al. [23] who investigated the relationship between each segment and the race performance during the 1996 Paralympic Games and reported that the mean clean swimming speed was related to the mean race speed (r ≥ 0.88) in all functional classification classes and strokes. They also reported that the mean turn (7.5 m turn-in and -out) and finish segment (the last 7.5 m of the race) speed were associated with the mean race speed (r > 0.63 and r > 0.61 for the turn and finish segment, respectively) in most of the strokes and classes, except the turn segment in SB9 breaststroke and the finish segment in SB9 breaststroke and S8 butterfly. In their study, the mean start segment speed exhibited significant correlations with the mean race speed only in 13 out of 24 events analyzed. However, despite many correlation coefficients between the non-clean-swimming speed and the mean race speed observed, Daly et al. [23] also showed that the correlations between the mean race speed and the start, turn and finish speed were almost none when a partial correlation analysis was conducted with controlling the effect of clean swimming speed. This suggests that the clean swimming segment is the primary determinant of Paralympic swimming race performance, regardless of the functional classes.

Another Paralympic race analysis study that covered wide ranges of classes and strokes was conducted in 2017 by Pérez-Tejero et al. [20], who analyzed the races using the individual-based method. They reported that swimmers with intellectual (S14), visual (S11–S13), or low physical impairment (S8–S10) showed similar start and turn distances (breakout distances) to those in non-impaired regional or national level swimmers reported by Veiga et al. [18]. They also showed that the start and turn distances were shorter in severe physical impairment groups (S2–S4 and S5–S7).

A study focusing on visually impaired swimmers was conducted by Daly et al. [17] who compared race patterns between Olympic swimmers and visually impaired athletes to assess the impact of the impairment on the race. They showed that the relative duration of the start, clean swimming, turn, and finish segment were identical between Olympic swimmers and those in Paralympic S12 (moderate visual impairment) and S13 (low visual impairment) classes. On the other hand, the S11 class (severe visual impairment) had a longer turn segment relative duration than the other three groups. This is reasonable as all swimmers in the S11 class are required to wear blackened goggles [34], meaning that all swimmers have to compete with no visual information and rely on a physical signal provided by their assistant (with a tapping device). Therefore, it is likely difficult for those swimmers to change their swimming direction as quickly as swimmers in the other groups.

Among the literature summarized in the present review, there were no other studies that focused on Paralympic races, and knowledge in Paralympic swimming races seems to be limited compared with non-impaired swimmers. For example, even though Daly et al. [23] reported the relationship between each segment and the total race performance, it is likely that race strategies are different at present due to a number of factors such as revisions in functional classification criteria and potential changes in race strategies (in a similar way to non-impaired swimmers’ races as discussed earlier in this review). Furthermore, the recent race analysis conducted by Pérez-Tejero et al. [20] only presented the distance of each individual segment, and the time and velocity information are lacking, as the researchers noted as a limitation. Paralympic swimming competitions have 14 different classes depending on swimmers’ impairment type and degree, and the classification rules are occasionally revised. Therefore, more frequent detailed Paralympic race analyses are required for coaches and swimmers to know the best race strategy under the regulation and for ensuring the validity of the classification system.

### 3.6. Race Analyses Outside Competitions

While most of the race analysis studies focused on races in real competitions, there were some studies that analyzed simulated races performed outside competitions. Tor et al. [27] observed changes in the start, clean swimming, and turn segment performance from a month before a National Championship to the time of the competition. Using statistical linear mixed modeling, the researchers investigated the impact of each segment performance improvement on the total race performance enhancement and reported that the start and clean swimming segment played an important role for the improvement of race performance in a short period of time before a competition.

In another study conducted by Morales and Arellano [11], a regression analysis was also used to model the effect of age on differences in segmental performances (in simulated 50 m freestyle) between the sexes. It was shown that boys started outperforming girls around the age of 12–13 at every segment (including finishing race time) except for the start in which the tendency occurred around the age of 10. It was unclear why the start segment exhibited a different tendency from the other segments, but one possibility might be related to the difference in jump ability between boys and girls. It has been reported that boys achieve higher squat jump height compared with girls around the age of 10–11 [35] or even earlier [36]. As the squat jump ability seems to be a determinant of the start performance [37], the difference in jump ability between sexes might be an explanation for the earlier start performance advantage in boys compared with the other segments. Nevertheless, another study showed that the sex difference in squat jump height only occurred at the age of 12 [38] and Morales and Arellano [11] did not investigate the difference in squat jump height. Therefore, further studies are necessary to assess the effect of both sex and age on race segment performances. For example, longitudinal studies with race analysis as well as anthropometric and strength investigations would be useful.

A recent study [4] employed a multi-camera system that consisted of six underwater and five above-water cameras to conduct a detailed two-dimensional (on horizontal-vertical plane) race analysis for simulated 100 m breaststroke races. A disadvantage of such a system is that only one swimmer can perform in one trial, which might affect the performance of the athletes due to a lack of opponents. On the contrary, the advantage is that one could assess variables that cannot be obtained with currently available race analysis systems used in competitions, such as temporal velocity data and vertical displacement of the swimmer. For example, the breaststroke study investigated not only segmental time, breakout distance, and the mean stroke frequency and length, but also the peak velocity and glide distance in the start and turn segment and the velocity, stroke length and frequency in the transition stroke. The breaststroke study did not focus on the underwater kinematics; however, a potential benefit of a race analysis outside competitions is the capability of assessing such information. In breaststroke, for example, analyzing the timing and velocity changes during a set of locomotive techniques (glide, pull-out motion, and transition kick and stroke) would be useful. Therefore, future studies should focus on detailed kinematic variables, including underwater kinematics, during a simulated race condition. Furthermore, combining physiological measurements (heart rate monitoring, post-race lactate and oxygen uptake assessment) with race analysis outside competitions would also be beneficial to better understand swimming performance characteristics that cannot be investigated during real competitions.

### 3.7. Limitation in the Current Literature Review

The current literature review summarized spatiotemporal race analyses conducted both in and outside competitions. All studies that analyzed races outside competitions conducted races close to an actual competition condition (e.g., starting the race with a diving start following an electric signal and performing the race with a comparable effort to competitions without exceeding 15 m underwater locomotion distance in each lap). However, it is probable that differences exist between the conditions due to the lack of (or a small number of) opponents or an audience and distinct athletes’ cognitive states. For example, Jones et al. [39] showed that elite swimmers had interpreted cognitive and somatic anxiety as being more facilitative of their performance compared with non-elite swimmers, which implies that race comparison between different levels of swimmers might be affected by not only their biomechanical skills but also such a cognitive difference. To our knowledge, relationships between mental and environmental (with and without opponents and/or an audience) factors and the total or different segments of the race are unknown. These potential relationships should be investigated in future studies, which would be beneficial for further understanding and interpretation of the race analysis studies, especially those conducted outside the competitions.

## 4. Conclusions

Researchers have analyzed swimming races by dividing the race into sub-segments (start, clean swimming, turn, and finish). However, the definition of each segment varied one to another that was particularly the case in the turn segment. The large variability probably reflects changes in swimming race strategy and rules occurred over the last decades as well as a lack of specific knowledge (such as swimming skills related to the approach to the wall). When applying fixed-distance segment methods, it is likely that different definitions are necessary depending on the swimming strokes and swimmers’ levels.

Race analysis has been conducted mostly during long course senior competitions, and the number of studies for short course and young swimmers’ races is limited. Many race analysis studies have focused on either 100 or 200 m races, and there is a lack of knowledge for 50 m, long-distance, individual medley, and relay events. Despite the necessity of frequent observation for Paralympic races, few studies on this topic have been conducted, which is also a current issue that researchers should beware. As data obtainable in real competitions are limited, race analyses outside competitions should be conducted more to investigate detailed kinematic and physiological factors in swimming races.

## Figures and Tables

**Table 1:** Segment definitions used in the reviewed literature.

Segment	Definition
Start	From the start signal until the swimmer’s head reaches the first 15 m point [3,4,6,7,13,16,17,27] From the start signal until the swimmer’s head reaches the first 10 m point [15,23] From the start signal until the swimmer’s head breaks the water surface after the underwater locomotion [7,18,19,20,21] From the start signal until the swimmer completes the first arm cycle after the breakout [25] Unclear [11]
(16 articles)	



Turn	15 m around (7.5 m before and after) the turn [7,13,14,15,16,17,23] From the last hand entry before the turn to the head breakout after the underwater locomotion [7,8,18,20,22] From the last head surfacing before the turn to the head breakout after the underwater locomotion (breaststroke) [20] From 5 m before the wall until the swimmer’s head reaches the 15 m point after the turn [3,6,12] From 5 m before the wall until the swimmer’s head reaches the 10 m point after the turn [4,27] From the wall contact to the head breakout after the underwater locomotion [19,21,24] From the last hand entry before the turn (front crawl only) or wall contact (the other three strokes) until the swimmer completes the first arm cycle [26] Unclear [11]
(21 articles)	






Finish	Last 10 m of the race [15] Last 7.5 m of the race [23] Last 5 m of the race [4,6,13,16,17] Unclear [11]
(8 articles)	


Clean swimming	The rest of the race [4,7,8,13,15,16,17,18,21,22,23,24,27] From 15 m to 35 m in each lap [12] Unclear [11]
(15 articles)	


**Table 2:** Breakout distance after the start in the four swimming strokes in 1984 and recent years in male events (the numbers in the table only show the mean value presented in each study).

Authors	Level	Race Distance	Male Freestyle	Male Backstroke	Male Breaststroke	Male Butterfly
* Miller et al. (1984)	International	100 m	9.14	8.51	11.10	9.89
		200 m	9.02	8.50	11.82	9.93
Veiga et al. (2014)	Regional	100 m	9.00	11.65	11.86	10.61
		200 m	8.95	10.87	12.02	10.62
	National	100 m	9.17	12.87	12.06	12.16
		200 m	9.21	11.73	12.42	11.59
Veiga et al. (2016)	International	100 m	10.47	13.88	13.44	13.82
		200 m	11.72	13.89	14.25	13.52
Morais et al. (2019)	International	100 m	11.43	12.34	13.02	12.53
** Marinho et al. (2020)	International	100 m	11.50	13.50	13.00	13.00
		200 m	12.00	13.50	14.50	12.00
Approximate increase in the breakout distance from 1984 to the present (%)		100 m	12.84	50.98	14.20	25.62
		200 m	18.36	49.39	12.00	22.32

* the distance was defined as the hand displacement where the swimmers completed the first stroke. ** displayed values are approximate as the precise numbers were not presented in the source.

**Table 3:** Breakout distance after the start in the four swimming strokes in 1984 and recent years in female events (the numbers in the table only show the mean value presented in each study).

Authors	Level	Race Distance	Female Freestyle	Female Backstroke	Female Breaststroke	Female Butterfly
* Miller et al. (1984)	International	100 m	8.12	7.66	10.44	8.87
		200 m	7.94	7.09	10.38	8.79
Veiga et al. (2014)	Regional	100 m	8.41	10.43	10.55	9.23
		200 m	8.50	9.22	10.57	9.04
	National	100 m	8.05	11.87	10.52	10.69
		200 m	8.48	10.74	10.84	10.18
Veiga et al. (2016)	International	100 m	10.74	13.51	11.91	-
		200 m	10.65	13.03	12.52	12.28
Morais et al. (2019)	International	100 m	10.74	12.85	11.37	11.54
** Marinho et al. (2020)	International	100 m	11.00	13.00	12.00	13.00
		200 m	10.00	13.00	13.00	13.00
Approximate increase in the breakout distance from 1984 to the present (%)		100 m	20.54	60.99	7.95	25.31
		200 m	22.49	66.40	13.55	30.83

* the distance was defined as the hand displacement where the swimmers completed the first stroke. ** displayed values are approximate as the precise numbers were not presented in the source.

**Table 4:** Breakout distance after the turn in the four swimming strokes in 1984 and recent years in male events (the numbers in the table only show the mean value presented in each study).

Authors	Level	Race Distance	Male Freestyle	Male Backstroke	Male Breaststroke	Male Butterfly
* Chow et al. (1984)	International	100 m	5.07	6.06	8.39	6.03
		200 m	4.93	6.04	-	6.17
Veiga et al. (2014)	Regional	100 m	7.00	9.12	10.01	8.17
		200 m	6.33	8.43	9.66	7.47
	National	100 m	7.04	11.06	9.97	9.66
		200 m	6.61	8.97	10.31	8.31
Veiga et al. (2016)	International	100 m	6.92	12.04	9.73	11.75
		200 m	7.09	10.25	10.25	9.51
Morais et al. (2019)	International	100 m	7.76	11.02	9.74	10.79
** Marinho et al. (2020)	International	100 m	8.00	12.00	10.00	11.00
		200 m	8.00	10.00	10.00	8.00
Approximate increase in the breakout distance from 1984 to the present (%)		100 m	44.85	82.31	17.88	70.38
		200 m	46.15	64.42	-	43.59

* the distance was defined as the head displacement where the swimmers completed the first stroke. ** displayed values are approximate as the precise numbers were not presented in the source.

**Table 5:** Breakout distance after the turn in the four swimming strokes in 1984 and recent years in female events (the numbers in the table only show the mean value presented in each study).

Authors	Level	Race Distance	Female Freestyle	Female Backstroke	Female Breaststroke	Female Butterfly
* Chow et al. (1984)	International	100 m	4.38	5.27	7.47	5.47
		200 m	4.13	5.37	7.27	5.21
Veiga et al. (2014)	Regional	100 m	5.53	8.08	8.61	6.61
		200 m	5.76	7.79	8.51	6.43
	National	100 m	5.66	9.11	8.48	7.68
		200 m	6.13	8.24	9.10	7.24
Veiga et al. (2016)	International	100 m	6.04	9.85	8.25	-
		200 m	5.53	7.82	8.55	7.61
Morais et al. (2019)	International	100 m	6.61	10.61	8.22	8.46
** Marinho et al. (2020)	International	100 m	5.00	10.00	8.00	9.00
		200 m	6.00	7.00	8.00	8.00
Approximate increase in the breakout distance from 1984 to the present (%)		100 m	31.69	80.83	11.27	45.11
		200 m	37.66	52.12	15.97	46.92

* the distance was defined as the head displacement where the swimmers completed the first stroke. ** displayed values are approximate as the precise numbers were not presented in the source.
